# Association of serum Ninjurin2 levels with neurologic damage and postherpetic neuralgia occurrence: an observational cohort study in chinese herpeszoster patients

**DOI:** 10.18632/oncotarget.17640

**Published:** 2017-05-04

**Authors:** Guozhuan Zhang, Yingjiao Sun, Lei Wang, Hui Tian, Lishuang Liang

**Affiliations:** ^1^ Department of Pain Management, Qilu Hospital of Shandong University, Jinan, Shandong, China; ^2^ Department of Thoracic Surgery, Qilu Hospital of Shandong University, Jinan, Shandong, China

**Keywords:** Ninjurin2, neurologic damage, herpes zoster, postherpetic neuralgia occurrence

## Abstract

**Objectives:**

Postherpetic neuralgia(PHN) is the most common complication of herpeszoster (HZ) infection. The study aimed to explore whether serum Ninjurin2 (for nerve injury-induced protein 2, NINJ2), a novel neurologic damage related protein, is associated with nerve injury and the occurrence of PHN.

**Results:**

Seventy-four of the eighty patients completed the study. On the 7th day of AHN, the patients had significantly higher values of NINJ2, cold-sense dispersion (ΔCS), warm-sense dispersion (ΔWS), cold-pain dispersion (ΔCP), heat-pain dispersion (ΔHP) and NRS score compared to controls. Six months after herpes, thirty four patients developed PHN. The values of serum NINJ2, ΔCS, ΔWS, ΔCP and ΔHP in PHN patients remained higher than in the controls and the patients who did not develop PHN. The PHN patients had significantly lower values of serumNINJ2 than patients who did not develop PHN in the period of AHN. With partial correlations and multivariable logistic regressions, serum NINJ2 level was strongly predictive of PHN.

**Materials and Methods:**

Eighty individuals with acute herpetic neuralgia (AHN) and 60 controls were recruited. The following nerve injury testing was performed on all of the patients including: the numerical rating scale (NRS) test, quantitative sensory testing (QST), and the investigation of serum NINJ2 levels on the 7th day of AHN; in addition, all of the patients repeated all of the items at 6 months after herpes.

**Conclusions:**

The serum NINJ2 levels are associated with nerve injury, particularly with nerve recovery, and may be predictors of PHN occurrence.

## INTRODUCTION

### Herpes zoster and postherpetic neuralgia

Zoster results from the reactivation of endogenous varicella zoster virus (VZV), which persisted in the latent form within sensory ganglia following varicella [1, 2]. Herpes zoster(HZ) typically begins with unilateral pain that persists for several days before the appearance of a rash [3]. It may manifest as throbbing pain, stabbing pain, burning pain, lancinating pain, and other types of pain, and is consistent with a skin lesion. The pain may also occur accompanied by the rash or after the lesion heals [4]. Acute herpetic neuralgia (AHN) is defined as pain that occurs within 30 days after rash onset [5]. Postherpetic neuralgia(PHN) is the most common complication and is usually defined as pain lasting beyond 6 months after the onset of zoster [6, 7].

### Epidemiology and mechanism of PHN

In this study, PHN was defined as neuralgia lasting ≥ 6 months [8]. Risk factors for PHN include increasing age, hyperglycemia, the presence of prodromal pain, the extent and severity of rash, and the severity of acute HZ pain [9, 10]. An age ≥ 50 years is more predictive of an occurrence of PHN if the patient was treated early with antiviral medications. There are other less replicated risk factors for PHN, such as female gender, location in the ophthalmic branch of the trigeminal nerve, greater neurosensory disturbance, and psychosocial distress [11, 12]. PHN can have a tremendous negative effect on quality of life due to its severe and durable pain. In population-based studies, zoster has been determined to be a risk factor for the development of major depression, and PHN has been reported to be the most common cause of suicide because of pain in persons aged 70 years and older [13]. The mechanism of PHN is still poorly understood. Thus, it is necessary to study the potential cause of PHN occurrence and to take related actions to prevent PHN.

### Ninjurin2 and nerve injury

Ninjurin2(for nerve injury induced protein 2, NINJ2) is a homolog of ninjurin1(for nerve injury induced protein 1, NINJ1), a homophilic cellular adhesion molecule, that was previously isolated as a gene induced in Schwann cells after nerve injury [14]. NINJ2 mRNA was detected at low levels in normal nerves but was highly upregulated after nerve injury and reached peak levels 7–14 days after injury [14]. NINJ2 is upregulated in Schwann cells in the distal nerve segment after peripheral nerve injury, and it promotes neurite outgrowth from dorsal root ganglion neurons (DRG neurons) via NINJ2-mediated homophilic cellular interaction between neuronal axons and Schwann cells [14].

The disease course from HZ to PHN is a continuous process, and nerve injury and nerve recovery runs through the course. Thus, we propose that NINJ2 may be involved in the occurrence of PHN, and may help to explore the mechanism and predict the occurrence of PHN.

## RESULTS

### Study population

Of the 80 patients enrolled, 74 patients completed the study. Reasons for withdrawal included heart attacks (*n* = 2), cerebrovascular disease emergency (*n* = 2), death (*n* = 1), and unexplained reasons (*n* = 1).

The clinical characteristics of the subjects in all groups are listed in Table 1. The patient group and controls showed no significant differences with regard to gender, age, DM, hypertension, or CAD (*p* > 0.05).

**Table 1:** Clinicopathological characteristics of the subjects

Variables	Controls (*n* = 60)	All Patients (*n* = 74)	Non-PHN patients (*n* = 40)	PHN patients (*n* = 34)
Male (%)	43.3	45.9	44.1	47.5
Age (year)	66.62 ± 8.281	66.14 ± 10.00	63.75 ± 9.00	68.94 ± 10.52^▲^
DM (%)	16.7	16.2	5	29.4^▲^
HP (%)	55	54	60	47.1
CAD (%)	58.3	52.7	47.5	58.8
NRS-7d	0.10 ± 0.04	5.35 ± 0.29^***^	5.82 ± 0.40^***^	4.95 ± 0.4^***^
ΔCS-7d	1.66 ± 0.09	6.12 ± 0.14^***^	6.29 ± 0.19^***^	6.09 ± 0.17^***^
ΔWS-7d	1.32 ± 0.08	5.05 ± 0.1^***^	4.86 ± 0.17^***^	5.21 ± 0.12^***^
ΔCP-7d	1.84 ± 0.12	3.44 ± 0.09^***^	3.5 ± 0.12^***^	3.39 ± 0.12^***^
ΔHP-7d	1.44 ± 0.09	2.17 ± 0.07^***^	2.25 ± 0.12^***^	2.10 ± 0.09^***^
NINJ2-7d (pg ml-1)	439.50 ± 77.84	1404.49 ± 151.47^***^	1969.97 ± 230.02^***^	739.21 ± 109.94^*▲ ▲^
NRS-6m	0.083 ± 0.28	3.35 ± 3.19^*^	0.68 ± 0.11^***^	6.5 ± 0.30^***^^▲ ▲^
ΔCS-6m	1.65 ± 0.69	3.7 ± 2.25^*^	1.79 ± 0.10	5.95 ± 0.13^***^^▲ ▲^
ΔWS-6m	1.35 ± 0.57	2.97 ± 1.77^**^	1.48 ± 0.08	4.72 ± 0.15^***^^▲ ▲^
ΔCP-6m	1.78 ± 0.7	2.37 ± 0.67^**^	1.97 ± 0.07	2.84 ± 0.10^***^^▲ ▲^
ΔHP-6m	1.43 ± 0.70	2.1 ± 0.66^**^	1.74 ± 0.08^*^	2.52 ± 0.10^***^^▲ ▲^
NINJ2-6m (pg ml-1)	420.47 ± 57.2	575.34 ± 43.74^*^	348.76 ± 33.47	841.91 ± 83.15^**▲ ▲^

Abbreviations: DM, diabetes mellitus; HP, hypertension; CAD, coronary heart disease; NRS, numerical rating scale; ΔCS, ΔWS, ΔCP, ΔHP, the differences of threshold temperature of cold sense, warm sense, cold pain and heat pain between the two sides of the corresponding; NINJ2, nerve injury induced protein 2. N/A : Not available.

Values are given as mean ± s.d.

*P < 0.05; **P < 0.01; ***P < 0.001 compared with controls.

^▲^*P* < 0.05; ^▲ ▲^*P* < 0.001 compared with non-PHN patients.

### Nerve injury and NINJ2 level testing

The ΔCS, ΔWS, ΔCP, ΔHP, NRS score, and serum NINJ2 levels were significantly greater in the patient group compared with the control group (*p* < 0.001, Table 1).

Thirty-four patients developed PHN at 6 months after the rash in our follow-up. Age and the blood glucose level on the 7th day after the rash were significantly higher in the PHN patients compared to patients who did not develop PHN (*p* < 0.05, Table 1 ), but the gender, percentages of hypertension and coronary heart disease of both the PHN patients and non-PHN patients were not significantly different from the control (*p* > 0.05). On the 7th day after the rash, the distributions of all types of temperature sensory data and the NRS score were not significantly different between the PHN patients and non-PHN patients (*p* > 0.05, Table 1), but both of these groups dramatically differed from the controls (*p* < 0.001, Table 1). The serum NINJ2 levels were significantly greater in PHN patients and non-PHN patients compared with the controls, with the highest levels observed in non-PHN patients (1969.97 ± 230.02, 739.21 ± 109.94 and 439.50 ± 77.84 pg ml-1, respectively, Table 1) on the 7th day after the rash.

Six months after herpes, the ΔCS, ΔWS, ΔCP, ΔHP, and NRS score of the PHN patients were greater than those of the controls and the non-PHN patients (*p* < 0.001, Table 1). However, for the non-PHN patients, the ΔCS, ΔWS, ΔCP decreased to normal levels, but ΔHP and the NRS score remained higher than in the controls (*p* < 0.05, *p* < 0.001, Table 1). The serum NINJ2 levels were significantly greater in PHN patients compared with controls and non-PHN patients (841.91 ± 83.15, 420.47 ± 57.2 and 348.76 ± 33.47 pg ml-1, respectively, Table 1 ).

**Figure 1:**
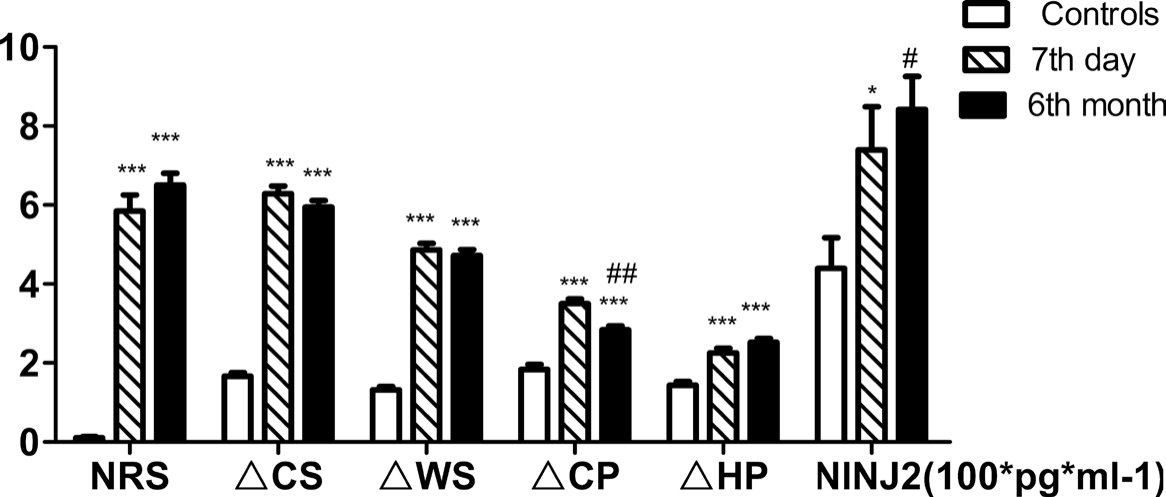
Changing of QST and serum NINJ2 levels of PHN patients Abbreviations: NRS, numerical rating scale; ΔCS, ΔWS, ΔCP, Δ HP, the differences of threshold temperature of cold sense, warm sense, coldpain and heat pain between the two sides of the corresponding; NINJ2, nerve injury induced protein 2. Values are given as mean ± s. d. *P < 0.05; **P < 0.01; ***P < 0.001 compared with control group; ^#^*P* < 0.05; ^##^*P* < 0.001 compared with 7th day; One hundred times of NINJ2 values this chart shows are the actual value.

**Figure 2:**
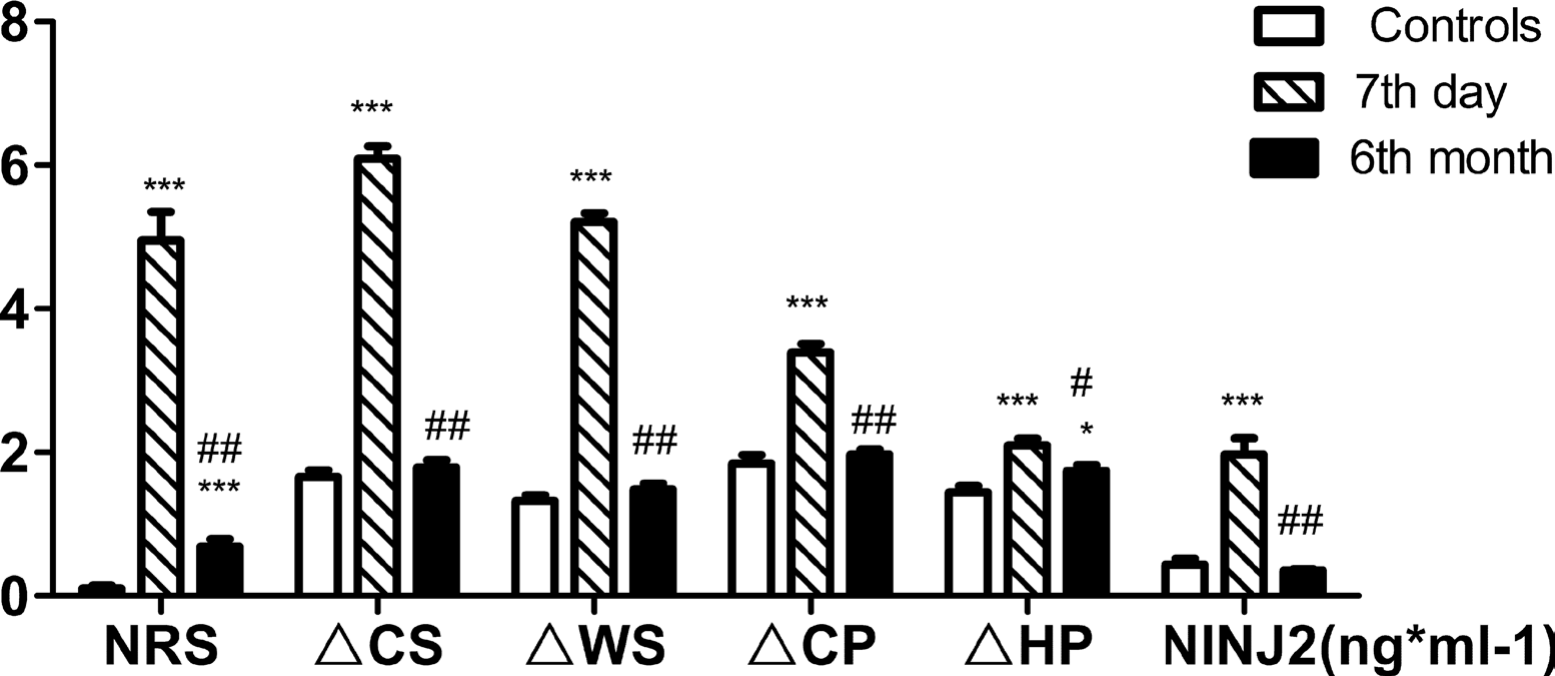
Changing of QST and serum NINJ2 levels of non-PHN patients Abbreviations: NRS, numerical rating scale; ΔCS, ΔWS, ΔCP, ΔHP, the differences of threshold temperature of cold sense, warm sense, coldpain and heat pain between the two sides of the corresponding; NINJ2, nerve injury induced protein 2. Values are given as mean ± s. d. *P < 0.05; **P < 0.01; ***P < 0.001 compared with control group; ^#^*P* < 0.05; ^##^*P* < 0.001 compared with 7th day; One thousand times of NINJ2 values this chart shows are the actual value.

For the PHN patients, the ΔCS, ΔWS, ΔCP, ΔHP, and NRS score were significantly greater than in the controls and were similar to those observed during AHN (*p* < 0.001, Figure 1). The serum NINJ2 levels of the PHN patients after 6 months were even greater than those on the 7th day after the rash (841.91 ± 83.15, 739.21 ± 109.94 pg ml-1,respectively, Figure 1). For the non-PHN patients, the ΔCS, ΔWS, ΔCP, ΔHP, NRS score, and serum NINJ2 levels were significantly greater than in the controls on the 7th day after the rash(*p* < 0.001, Figure 2), ΔHP and NRS score maintained still higher levels than controls after 6 months (*p* < 0.001, *p* < 0.05, Figure 2) whereas ΔCS, ΔWS, ΔCP and serum NINJ2 levels returned to normal levels after 6 months compared with controls (*p* > 0.05, Figure 2).

### Correlation of PHN occurrence with clinical parameters and NINJ2

In all patients, after controlling for gender, hypertension, and coronary heart disease, PHN occurrence showed significant correlations with the lower serum NINJ2 levels, presence of diabetes and older age on the 7-th day (*r* = −0.404, *p* < 0.001; *r* = 0.277, *p* = 0.022; *r* = 0.279, *p* = 0.018), but does not appear to be correlated with NRS-7d, ΔCS-7d, ΔWS-7d, ΔCP-7d and ΔHP-7d (*p* = 0.110–0.686) (Table 2). The higher NINJ2-6m level was significantly correlated with older age at baseline and higher NRS score, bigger ΔCS, ΔWS, ΔCP and ΔHP (*r* = 0.283, *p* = 0.019; *r* = 0.641, *p* < 0.001; *r* = 0.602, *p* < 0.001; *r* = 0.611, *p* < 0.001; *r* = 0.398, *p* = 0.001; *r* = 0.509, *p* < 0.001, respectively) (Table 3). In the multivariable logistic regression analysis adjusting for serum NINJ2 levels on the 7-th day after rash, age, and blood glucose, all of them were significantly associated with PHN occurrence (β = −0.001, *p* < 0.001; β = 0.055, *p* = 0.029; β = 2.069, *p* = 0.011) (Table 4). In other word, the log odd of PHN occurrence increases 0.055 for one-year older patient, decreases 0.001 for one unit (pg/ml) increase of NINJ2 levels on the 7-th day after rash, or is 2.069 bigger in patients with diabetes than that in patients without diabetes, when adjusting for the other variables.

**Table 2:** Partial correlations analysis between NINJ2 levels, PHN occurrence and parameters at the 7th day of herpes

	NINJ2-7d		PHN occurrence	
	Partial coefficient	*p* value	Partial coefficient	*p* value
Age(year)	0.067	0.575	0.279	0.018
DM	−0.05	0.0627	0.277	0.022
NINJ2-7d			−0.404	< 0.001
NRS-7d	0.093	0.44	0.168	0.162
ΔCS-7d	−0.13	0.28	0.093	0.441
ΔWS-7d	−0.049	0.686	−0.191	0.11
ΔCP-7d	−0.096	0.425	0.091	0.448
ΔHP-7d	−0.078	0.52	0.119	0.325

Abbreviations: DM, diabetes mellitus; NINJ2, nerve injury induced protein 2, PHN, postherpetic neuralgia.

**Table 3:** Partial correlations analysis between NINJ2 levels and parameters after 6 months

	NINJ2	
	Partial coefficient	*p* value
Age(year)	0.283	0.019
NRS-6m	0.641	< 0.001
ΔCS-6m	0.602	< 0.001
ΔWS-6m	0.611	< 0.001
ΔCP-6m	0.398	0.001
ΔHP-6m	0.509	< 0.001
PHN	0.707	< 0.001

Abbreviations: NRS, numerical rating scale; ΔCS, ΔWS, ΔCP, ΔHP, the differences of threshold temperature of cold sense, warm sense, cold pain and heat pain between the two sides of the corresponding; NINJ2, nerve injury induced protein 2; PHN, postherpetic neuralgia.

**Table 4:** Analysis and selection of variables affecting the PHN occurrence in multivariable logistic regression model

Dependent variable	Independent variables	β	*P*-value
PHN	Age (year)	0.055	0.029
PHN	DM	2.069	0.011
PHN	NINJ2-7d	−0.001	< 0.001

Abbreviations: DM, diabetes mellitus; NRS, numerical rating scale; NINJ2, nerve injury induced protein 2; PHN, postherpetic neuralgia.

β: standard regression coefficient.

## DISCUSSION

Previous studies have found that NINJ2 is associated with nerve injury in ischemic stroke [15]. Toshiyuki Araki and Jeffrey Milbrandt [14] found that NINJ2 is expressed at low levels in the normal nerve but is highly upregulated after nerve injury. NINJ2 is associated with nerve injury and nerve recovery. The disease course from HZ to PHN is a continuous process, and nerve injury and nerve recovery run through this course. Thus, we detected the relationship between serum NINJ2 levels and PHN occurrence. Our current study is the first to report associations between serum NINJ2 levels and the parameters of nerve injury and nerve recovery and to detect the relationship between serum NINJ2 levels and PHN occurrence.

In our study, serum NINJ2 levels increased to different degrees during AHN and PHN. The serum NINJ2 levels were significantly greater in PHN patients and non-PHN patients compared with the controls, accordance with the ΔCS, ΔWS, ΔCP, ΔHP, NRS score, with the highest NINJ2 levels observed in non-PHN patients on the 7th day after the rash. During AHN, nerve injury occurred and the body began responding to this injury and nerve recovery began. Six months after herpes, for the PHN patients, the serum NINJ 2 levels remained higher the controls and even higher than those detected during AHN. However, for the non-PHN patients, the serum NINJ2 levels decreased to normal contrary to the ΔCS, ΔWS, ΔCP. The ΔHP and NRS scores of non-PHN patients were more or less higher than control, but the NRS kept under 1. When PHN occurred, nerve injury and nerve recovery continued and lased for 6 months. But when HZ and nerve injury cured, nerve recovery slowed down to the normal condition. Accordantly, the serum NINJ2 levels were significantly greater in PHN patients compared with controls and non-PHN patients (841.91 ± 83.15, 420.47 ± 57.2 and 348.76 ± 33.47 pg ml-1, respectively, Table 1). So NINJ2 levels were significantly correlated with clinical markers of nerve injury and nerve recovery during the process of PHN. Taken together, these findings suggested that serum NINJ2 levels may predict the occurrence of PHN

In our study, serum NINJ2 levels increased to different degrees during AHN and PHN. NINJ2 levels were significantly correlated with clinical markers of nerve injury and nerve recovery during the process of PHN. Taken together, these findings suggested that serum NINJ2 levels can predict the occurrence of PHN. Activation of latent Varicella zoster virus (VZV) causes generalized necrosis and cell death in the skin and within the nerve, root, and ganglion [16]. Previous studies also demonstrated damage in the dorsal root ganglia (DRG) as well as a loss of neurons in the affected spinal nerves and dorsal horn [17, 18]. Nerve recovery occurs immediately following injury, but in human studies on sensory nerve injuries and their recovery, the precise pathophysiological mechanism and the extent of nerve injury are not known due to the heterogeneity of patients with regard to the type, degree, and age of the lesion, all of which affect recovery [19, 20]. Because acute nerve damage is widespread in patients, no relationship was observed in the partial correlation analysis of the relationship between NINJ2 levels and the parameters of nerve injury on the 7th day. However, after 6 months, partial correlation analysis showed that higher serum NINJ2 levels were significantly correlated with older age, higher NRS score, bigger ΔCS, ΔWS, ΔCP, and ΔHP (*r* = 0.283, *p* = 0.019; *r* = 0.641, *p* < 0.001; *r* = 0.602, *p* < 0.001; *r* = 0.611, *p* < 0.001; *r* = 0.398, *p* = 0.001; *r* = 0.509, *p* < 0.001) (Table 3). Thus, serum NINJ2 levels were correlated with nerve injury and particularly with nerve recovery.

Following acute zoster, the patients were characterized with dermatomal areas of cutaneous scarring and sensory loss independent of PHN [21, 22]. The anatomical loss and injury of afferent neurons and fibers were consistent with the functional decrease in sensory perception within the affected dermatomes. In our study, the values of all types of temperature sensory data and the NRS score increased compared with controls, which indicated that peripheral sensory nerves suffered extensive damage. After 6 months, the ΔCS, ΔWS, ΔCP, and serum NINJ2 levels of the non-PHN patients significantly decreased, but ΔHP remained higher than normal. For the PHN patients, ΔCP decreased but remained higher than normal, and the ΔCS, ΔWS, ΔHP, and NRS score of the PHN patients remained significantly higher than in the control group. The serum NINJ2 levels of the non- PHN patients increased significantly compared to the levels observed on the 7th day (*p* < 0.05), which suggested that NINJ2 levels were related with nerve recovery and repair.

Excess electrical activity of damaged peripheral nociceptive neurons is a major cause of acute zoster pain and of inflammatory and nociceptive pain in general. If the pain continues, then the electrical responses of dorsal horn nociceptive neurons that project to the brain may increase and cause sensitization, and PHN can occur. The important features of pain following nerve injury can be discerned from clinical observation [23]. In our study, 46 percent of HZ patients developed PHN. The well-defined risk factors for PHN in patients with HZ include older age, a high blood glucose level, presence of prodromal pain, the extent and severity of rash, and the severity of acute HZ pain [9]. Specifically, both genetic and environmental influences contribute to the likelihood of developing PHN after nerve injury during AHN [23]. In our study, we found that the occurrence of PHN is significantly correlated with age, blood glucose level and serum NINJ2 levels using partial correlation analysis and multivariable linear regression analysis (Table 2, Table 4).

In this study, we found that serum NINJ2 levels were correlated with nerve recovery.NINJ2 is associated nerve repair when nerve system facing injury. In the early stages of the disease, more NINJ2 means more protection and potential repair for non- PHN subjects. HZ patients may not develop PHN when they exhibit high levels of serumNINJ2 during a AHN. In the patients who developed PHN, nerve recovery continued and the serum NINJ2 level remained relative high for a long time. These results suggest that serum NINJ2 levels may be a predictor of PHN in early stage.

A potential limitation of the present study was that prospective studies should provide further insight into the potential cause–effect relationships between preclinical markers and NINJ2 levels. There may be several other factors affecting the association between clinical markers and NINJ2, which requires additional studies.

## MATERIALS AND METHODS

### Participants

This research was approved by the Ethics Committee of Qilu Hospital. A total of 80 patients who were diagnosed with AHN within 7 days at the Department of Pain Management, Qilu Hospital, from January 2014 to June 2015, were enrolled for our research. 60 volunteers who were recruited as controls at the Department of Pain Management, Qilu Hospital, from January 2014 to June 2015. For all volunteers, neurological and routine blood parameters were assessed to meet the exclusion criteria. The research ethics boards at the participating centers approved the study protocol, and each participants were informed and provided their written informed consent before participating in the study. The study was performed according to the Helsinki Declaration.

Inclusion criteria for HZ patients were as follows: 1) Provided written informed consent; 2) ≥ 50 years of age; 3) diagnosed with HZ within 7 days; 4) sufficient cognitive ability to be able to accurately assess their own state, including pain intensity, quality of life, and adverse reactions; and 5) no other form of neuralgia or nerve injury condition; Inclusion criteria for controls were as follows: 1) Provided written informed consent; 2) ≥ 50 years of age; 3) sufficient cognitive ability to be able to accurately assess their own state, including pain intensity, and quality of life; 4) no form of neuralgia or nerve injury condition; and 5) an NRS score of 0–1.

Exclusion criteria were as follows: 1) Severe disease of the liver or other organs; 2) a mental disorder that prevented them from evaluating themselves accurately; 3) any form of neuralgia or nerve injury condition; and 4) cerebral stroke or other form of cerebrovascular disease.

### Study design

All of the participants were given a numerical rating scale (NRS) test, quantitative sensory testing (QST), and a 2-ml blood draw to test NINJ2 levels on the day of enrollment for the controls and on the 7th day of HZ for the patients. Six months after the rash, All patients repeated all of the items 6 months after the rash. The volunteers also were given same testing 6 months after the enrollment.

When enrolled, all the patients were given oral acyclovir ( Bangna, Xinda, Shandong, China) for 7 days, and analgesic therapies, including oral pregabalin (Lyrica, Pfizer, NY, America) and oxycodone (OxyContin, Mundipharma, Beijing, China). The start dosage of pregabalin was 150mg/day, and if there was no observed change of NRS, the dosage of pregabalin was increased to 300 mg/day, until the maximal dose 600 mg/day, and the start dosage oxycodone was 20 mg/day, and the dosage increased according to pain assessment. If patients appeared adverse events including dizziness, somnolence, vertigo, dry mouth, constipation, edema, and so on, we would stop adding dosage and given corresponding therapies. The patients were administered antiviral and analgesic therapies during the intervening period. Two independent experts made the diagnosis of PHN according to the symptoms, characteristics of pain, QST and NRS score.

### Outcome measures

#### Baseline clinical characteristics

The gender, age, history of illness (such as hypertension), and medication history were recorded.

### Pain intensity

Pain intensity was assessed using NRS before study participation and at the follow-up visits with 0 = no pain and 10 = the worst pain imaginable. Patients dictated or lined out the score, which could describe their pain density.

### QST

HZ and PHN involve nerve injury, and neuropathic pain (NP) is the predominant manifestation of the nerve injury. We diagnosed nerve injury and PHN based on QST in addition to neuropathic pain symptoms and signs. QST is a noninvasive procedure that is helpful in the assessment of the function of small Aδ and C nerve sensory fibers, which is hardly evidenced by other methods [24, 25]. The assessments were performed in a quiet, temperature-controlled room (mean across all tests: 23.4°C) with a computerized thermal sensory analyzer (TSA) model TSA-II (Medoc Inc., Ramat Yishai, Israel). QST was performed at two sites on the body: (1) the affected side and (2) the contralateral side (the mirror region). We detected cold-sense (CS), warm-sense (WS), cold-pain (CP) and heat-pain (HP) thresholds using the method of limits. Subjects were instructed to press are sponse button when a thermal sensation (either cold or warm) was first perceived for the CS and WS processes, which started from a resting neutral temperature of 32°C and gradually decreased or increased by 1°C per second [25, 26]. Next, the computer recorded the threshold temperature and returned to the neutral temperature. The procedure was similar for CP and HP, but the subjects may feel discomfort for these tests and they were instructed to press the response button immediately after they perceived the thermal sensation as painful. The differences in threshold temperature between the two sides of the body were recorded as the dispersions of QST for comparison and were recorded as ΔCS, ΔWS, ΔCP, ΔHP.

### Serum NINJ2 levels detected using enzyme-linked immune sorbent assay (ELISA)

Serum NINJ2 levels were determined using an ELISA kit (Proteintech, Rosemont, USA) according to the manufacturer's instructions. The absorbance values were measured at 450 nm [27].

### PHN

Two experts diagnosed that the patients were suffering from PHN 6 months after the rash. PHN is primarily a clinical diagnosis. A history of herpes zoster rash, followed by persistent pain (include throbbing pain, stabbing pain, burning pain, lancinating pain, and other types of pain) in the corresponding dermatome or adjoining area is typical [28].

### Statistical analysis

Continuous data are presented as the mean ± SD. Categorical data were summarized as percentages. One-way ANOVA of normally distributed continuous data was used to compare the differences among groups of subjects. Comparison of the prevalence of comorbid conditions were made using the χ^2^ test or Fisher's exact test if necessary. The correlation between two variables was assessed by Pearson or Spearman correlation coefficient analysis. After controlling for covariates, bivariate correlations underwent partial correlation analysis. Multivariable logistic regressions using the forward conditional method were used to determine the variables that were putative predictive factors (As mentioned before, age and diabetes are independent risk factors confirmed by other studies [10, 11]). So age, diabetes and NINJ2-7d were added into multivariable logistic regression analysis as independent variables when PHN occurrence was dependent variable). All statistical analyses were performed using SPSS 12.0 software (SPSS Inc. Chicago, Illinois, America). *P*-values < 0.05 were considered statistically significant.

## CONCLUSIONS

In conclusion, the serum NINJ2 level is associated with nerve injury, and with nerve recovery in particular, and may be a predictor of PHN occurrence.
